# Including mixed methods research in systematic reviews: Examples from qualitative syntheses in TB and malaria control

**DOI:** 10.1186/1471-2288-12-62

**Published:** 2012-04-30

**Authors:** Salla Atkins, Annika Launiala, Alexander Kagaha, Helen Smith

**Affiliations:** 1 Division of Global Health, Karolinska Institute, Stockholm, Sweden; 2 Institution of Public Health and Clinical Nutrition, University of Eastern Finland, Kuopio, Finland; 3 Department of Sociology, Makerere University, Kampala, Uganda; 4 International Health Group, Liverpool School of Tropical Medicine, Liverpool, UK

## Abstract

**Background:**

Health policy makers now have access to a greater number and variety of systematic reviews to inform different stages in the policy making process, including reviews of qualitative research. The inclusion of mixed methods studies in systematic reviews is increasing, but these studies pose particular challenges to methods of review. This article examines the quality of the reporting of mixed methods and qualitative-only studies.

**Methods:**

We used two completed systematic reviews to generate a sample of qualitative studies and mixed method studies in order to make an assessment of how the quality of reporting and rigor of qualitative-only studies compares with that of mixed-methods studies.

**Results:**

Overall, the reporting of qualitative studies in our sample was consistently better when compared with the reporting of mixed methods studies. We found that mixed methods studies are less likely to provide a description of the research conduct or qualitative data analysis procedures and less likely to be judged credible or provide rich data and thick description compared with standalone qualitative studies. Our time-related analysis shows that for both types of study, papers published since 2003 are more likely to report on the study context, describe analysis procedures, and be judged credible and provide rich data. However, the reporting of other aspects of research conduct (i.e. descriptions of the research question, the sampling strategy, and data collection methods) in mixed methods studies does not appear to have improved over time.

**Conclusions:**

Mixed methods research makes an important contribution to health research in general, and could make a more substantial contribution to systematic reviews. Through our careful analysis of the quality of reporting of mixed methods and qualitative-only research, we have identified areas that deserve more attention in the conduct and reporting of mixed methods research.

## Background

Health policy makers now have access to a greater number and variety of systematic reviews to inform different stages in the policy making process, including reviews of qualitative research. Systematic reviews of observational studies help identify the magnitude of a particular health problem, reviews of randomized controlled trials provide reliable information the benefits or harms of policy options being considered, and reviews of economic evaluations help determine cost-effectiveness of different options [1]. Another form of evidence summary are systematic reviews of qualitative research (see eg. refs [2-4]). Which can be central to exploring patient or user views or experiences of a policy option, understanding health behaviours in relation to an illness or patient decision making and uptake of an intervention, as well as identifying barriers and facilitators to implementing specific interventions. Unlike the more established methods for reviewing effectiveness studies, methods for systematically reviewing qualitative research are still in development.

An important question in qualitative research synthesis is what type of study to include. Review authors might include studies where qualitative research is the main focus or where qualitative methods are combined with quantitative in the same study (mixed methods). Researchers tend to use mixed methods for pragmatic reasons, since the design allows for use of a range of methods and can yield more comprehensive findings [5,6]. Of course researchers use mixed methods designs for strategic reasons too. Key funders of health and development research recognize the value of combining qualitative and quantitative research, increasingly call for the inclusion of qualitative methods, and increased inter-disciplinary and social science contribution to proposals.

As the number of mixed methods studies increases, their inclusion in systematic reviews is more likely. But there are recognised design issues with mixed methods research that pose challenges for including and appraising these studies in systematic reviews. For example, in some cases the author’s intended rationale for using mixed methods may not match how they actually combined methods in practice, and the risk here is that the study produces unnecessary or redundant data which fails to address the research question [7]. There are also inherent problems with reporting findings of mixed methods research, particularly the role and sequence of different data collection methods and integration of analysis and findings [6-8].

The quality and utility of a systematic review depends on the quality of the included studies, and so the methodological challenges in mixed methods research are important for synthesis [9]. For reviews of effects, there are strict criteria for inclusion, and stringent criteria for assessing risk of bias in included studies [10]. In reviews that include qualitative and other research designs, decisions on which studies to include, and whether and how to conduct quality assessment are still widely debated [11,12]. Recent developments include a scoring system for appraising the methodological quality of mixed methods studies alongside qualitative and quantitative studies included in ‘mixed studies reviews’ [13] and guidelines for good reporting of mixed methods studies [7]. Even when time and effort is invested in quality appraisal systematic review authors rarely comment on the credibility, rigour of conduct or the contribution the different studies make to the synthesis outcome. Sometimes reviewers indicate that poorer quality studies contribute less to the synthesis, without further elaboration of the types of studies that were considered poorer quality and why. Yet scrutiny of the poorer quality studies might help determine which types of studies it is meaningful to summarise together and which could reasonably be excluded or summarised in other sorts of reviews.

Though there are indications that mixed methods studies tend to be poorly reported and fare poorly in methodological quality appraisal [14], we could not find any systematic analysis comparing the quality of reporting of qualitative-only research with qualitative research from mixed methods studies. In this exploratory study we assess how the quality of reporting and rigor of qualitative-only studies compares with that of mixed-methods studies. To do this we examined studies included in two completed systematic reviews - one focusing on factors influencing adherence to tuberculosis treatment [15] and the other on uptake of malaria preventive interventions in pregnancy [16]. Secondary objectives were to determine any differences in the quality of reporting between the malaria and TB focused studies and to explore any differences in the quality of reporting in qualitative-only and mixed methods studies over time.

## Methods

We used two completed systematic reviews [15,16] to generate a sample of qualitative studies and mixed method studies in order to make an assessment of how the quality of reporting and rigor of qualitative-only studies compares with that of mixed-methods studies.

### Description of studies

We compiled a database of included studies from each systematic review and for each study we recorded author name, year of publication, category of qualitative study (mixed methods or standalone), research design, and components of methodological quality, findings and recommendations of the studies (see additional file 1). We used this as the basis of our comparative analysis.

### Comparative analysis of quality and rigour

The studies in our sample had already been subject to quality assessment as part of the systematic review process (based on [17-19]) and we used the outcomes of this for our analysis. For this comparative analysis we chose to focus on the following criteria: description of rigour in conduct, description of study context, description of the method of analysis, and credibility and depth and richness of findings (Table 1). To assess rigour in this comparative analysis, we chose to combine four items to provide a ‘composite’ score of how well the research conduct was reported in the papers. We assessed whether or not each paper reported the following components: a clearly defined research question, rationale for the study design, a well defined sampling strategy and clear description of data collection. For each of these aspects a paper could score 1 if it reported the aspect or 0 if not.

**Table 1:** Quality criteria used in the comparative analysis

**Domain**	**Criteria explanation**	**Indicative questions**
Rigour in research conduct	Judgement on how carefully the research is carried out; tends to be a judgement of reporting quality	Is the research question clearly defined? Rationale for the study design discussed? Is a sampling strategy well defined and justified? Is the method of data collection clearly described?
Study context	A detailed description is needed to judge wider applicability of the findings; refers to transferability	Detailed description of the context of the study to allow assessment of applicability to other settings? Discussion of limits to wider inference?
Analysis procedure	An important component of rigour and reliability	Is the method of analysis clearly described?
Credibility	Judgement on how well the findings are presented and how meaningful or believable they are	How credible are the findings? Are the claims made supported by sufficient evidence?
Depth, detail & richness of findings	An indication of the quality of the analysis which underlies credibility claims	E.g. “thick vs. thin description”? Illumination of multiple perspectives/contribution of sample design? Detection of underlying factors/influences or conceptual linkages? Presentation of illuminating extracts/observations?
Contribution to knowledge	Judgement on the relevance and potential utility of the findings in relation to policy, practice or theory	Clear discussion of how the research findings contribute to: Understanding of uptake of malaria preventive interventions by pregnant women?; theoretical conceptions of uptake of malaria preventive interventions in pregnancy? New areas of investigation identified?

Criteria relating to study context and description of analysis procedure are also judgements on the quality of reporting, and for this analysis papers were assessed based on whether they provided detailed description of study context and of analysis procedures. The other criteria represent judgements on the believability of the findings (credibility) and whether the findings represent thick description. Recommendations for further research and policy and practice were extracted verbatim from each article. Policy and practice recommendations were coded “health education only”, “health education and intervention” “intervention only” and “policy only”.

Since methods for conducting mixed methods research are fairly new compared to the more established methods for qualitative research, we conducted an analysis of the quality of reporting over time to determine any improvements in the quality of reporting in these studies. We did this by organising the included studies into two time fames – we classified studies published between 1985–2003 as ‘older’ studies, and those published between 2004–2010 as ‘newer’ studies. We based this classification on the range of study publication years in our sample. With the exception of one study published in 1985, the range was 1992–2010. We explored any differences between older and newer studies in terms of the quality of reporting and rigor.

## Results

Overall our sample contained 40 qualitative-only studies (n = 21 TB review; n = 19 malaria review) and 38 mixed methods studies (n = 14 TB review; n = 24 malaria review) (Additional file 1). For the qualitative-only articles, the most commonly used data collection methods were interviews alone (10/21 TB focused studies) and a combination of focus group discussions (FGDs) and interviews (17/19 malaria studies). For mixed methods studies, interviews were commonly used alongside quantitative data collection methods (7/14 TB studies) a combination of FGDs and interviews were used alongside surveys in the malaria focused studies (12/24). The largest sample among the qualitative-only studies was in a study that used 823 semi-structured exit interviews. For mixed methods studies often qualitative data were obtained using interviewer-administered surveys with open ended questions integrated and this resulted in sample sizes of 200-300 participants and in one case 857 households.

### Quality assessment

Overall, the reporting of qualitative studies in our sample was consistently better when compared with the reporting of mixed methods studies (Table 2). Overall 65% of the articles reporting qualitative research and 32% of those reporting mixed methods research were rated ‘credible’. In relation to study context, more than half of the qualitative-only studies (58%) and the mixed methods studies (53%) provided sufficient description of context to judge wider applicability of the findings. Overall, studies in our sample did not report on research conduct well. Four mixed methods studies (11%) scored the highest possible rating, “4” for rigour, compared with eight (20%) qualitative articles. Overall 15 articles reporting qualitative research achieved a rigour score of 3, compared with 12 articles reporting mixed methods research. Generally more qualitative studies (45%) described the approach to analysis compared with mixed methods articles (26%). More qualitative studies (43%) provided thick description, or demonstrated depth and richness in the findings compared with mixed methods papers (18%) where reports represented thin or superficial description of qualitative data.

**Table 2:** Percentage of studies meeting the quality assessment criteria, by research design and by topic

	**Credibility (“yes”)**	**Description of context (“yes”)**	**Rigour in conduct (score 4)**	**Description of analysis (“yes”)**	**Depth detail and richness (“yes”)**
Qualitative studies					
TB review (n = 21)	13 (62%)	12(57%)	7 (33%)	8 (38%)	10 (48%)
Malaria review (n = 19)	13 (68%)	11 (58%)	1 (5%)	10 (53%)	7 (37%)
Total (n = 40)	26 (65%)	23 (58%)	8 (20%)	18 (45%)	17 (43%)
Mixed methods studies					
TB review (n = 14)	4 (29%)	3 (21%)	2(14%)	1 (7%)	1 (7%)
Malaria review (n = 24)	8 (33%)	17 (71%)	2 (8%)	9 (38%)	6 (25%)
Total (n = 38)	12 (32%)	20 (53%)	4 (11%)	10 (26%)	7 (18%)

We specifically explored any differences in the quality of reporting between the malaria and TB focused studies. For both the TB and malaria focused studies the overall trend was towards better reporting of research conduct, study context and analysis as well as more credible and rich findings in the qualitative-only articles compared with mixed methods articles. However, reporting of analysis methods and research context was particularly poor in mixed methods articles on TB adherence (7%) compared with malaria focused articles (38%) and the TB articles were less likely to provide rich findings. We also found that reporting of research conduct was better in the TB focused qualitative-only articles (33%) compared with the malaria focused qualitative-only articles (5%).

Our time-related analysis indicates that in both qualitative and mixed methods studies, newer studies are more likely to report on research context, describe analysis procedures, and be judged credible and provide rich data (see Table 3). However, an interesting finding is that reporting of research conduct in mixed methods studies did not appear to have improved over time in our sample. The time-related analysis also indicates that for older mixed methods studies the quality of reporting of analysis procedures and study context, and judgements of credibility and data richness, was worse than for the older qualitative studies. This trend is also reflected in the newer mixed methods studies where the reporting of research conduct and analysis, and the credibility and richness of findings, are worse compared to qualitative studies. One exception to this trend is that more of the newer mixed methods studies (74%) report on study context than the newer qualitative studies (68%).

**Table 3:** Quality assessment of mixed methods and qualitative papers by year of publication

	**Credibility (“yes”)**	**Description of context (“yes”)**	**Rigour in conduct (score 4)**	**Description of analysis (“yes”)**	**Depth detail and richness (“yes”)**
Qualitative studies					
1985-2003 (n = 18)	11 (61%)	8 (44%)	3 (17%)	3 (17%)	7 (39%)
2004-2010 (n = 22)	15 (68%)	15 (68%)	5 (23%)	15 (68%)	10 (45%)
Total (n = 40)	26 (65%)	23 (58%)	8 (20%)	18 (45%)	17 (43%)
Mixed methods studies					
1985-2003 (n = 19)	5 (26%)	6 (32%)	2 (11%)	3 (16%)	3 (16%)
2004-2010 (n = 19)	7 (37%)	14 (74%)	2 (11%)	7 (37%)	4 (21%)
Total (n = 38)	12 (32%)	20 (53%)	4 (11%)	10 (26%)	7 (18%)

### Recommendations made by mixed methods and qualitative articles

Health education, or health education together with specific programmatic interventions, were common recommendations made by authors of all studies included in our sample (Table 4). Similar numbers of qualitative studies (10%) and mixed methods studies (11%) recommended only health education. More qualitative papers recommended a combination of health education and specific programme intervention (53%) than mixed methods studies (34%). Both types of studies were less likely to indicate policy recommendations, while mixed methods studies were more likely to recommend only programme interventions. Similar numbers of mixed methods studies (42%) and qualitative studies (40%) made recommendations for further research. In the TB review, two qualitative articles did not make recommendations for policy or practice, while all mixed methods articles made recommendations.

**Table 4:** Recommendations for practice and policy from mixed methods and stand-alone qualitative papers

	**Health education only**	**Health education/ Intervention**	**Policy recommendations**	**Intervention only**	**No recommendations**	**Research implications**
Qualitative studies						
TB review (n = 21)	2 (10%)	9 (43%)	2 (10%)	6 (29%)	2 (10%)	9 (43%)
Malaria review (n = 19)	2 (11%)	12 (63%)	0 (0%)	4 (21%)	0 (0%)	7 (37%)
Total (n = 40)	4 (10%)	21 (53%)	2 (5%)	10 (25%)	2 (5%)	16 (40%)
Mixed methods studies						
TB review (n = 14)	2 (14%)	3 (21%)	0 (0%)	8 (57%)	0 (0%)	7 (50%)
Malaria review (n = 24)	2 (8%)	10 (42%)	2 (8%)	6 (25%)	0 (0%)	9 (38%)
Total (n = 38)	4 (11%)	13 (34%)	2 (5%)	14 (37%)	0 (0%)	16 (42%)

## Discussion

### Summary of findings

Using a small sample of studies we compared the quality of reporting of qualitative-only studies with that of mixed-methods studies, and found that mixed methods studies are less likely to provide a description of the research conduct or qualitative data analysis procedures and less likely to be judged credible or provide rich data and thick description compared with standalone qualitative studies. We found that in mixed methods articles on TB adherence, reporting of analysis methods and research context was poor and findings were less likely to be judged rich compared with mixed methods articles focused on malaria. Mixed methods papers published since 2003 are more likely to report on research context and analysis, and more new studies were judged to be credible and provide rich findings, but reporting of research conduct had not improved over time in our sample. Mixed methods and standalone qualitative studies made similar policy and practice recommendations and both designs infrequently identified areas of further research.

### Limitations in the comparative analysis

There are some limitations that affect the conclusions we can draw from this analysis. The TB review was conducted some years previously, so the quality assessment criteria differed slightly. In addition, the quality assessors were not the same in the TB review and the malaria review, although one author of this analysis was an author on both reviews. In both reviews, employing the criteria relating to rigor in conduct, description of context and analysis became an exercise in judging the quality of the written report as opposed to the quality of actual research procedure; a more detailed discussion of the implications of this is provided elsewhere [14]. For example, mixed methods papers might be reported less fully in journals with limited word counts as they would need to describe both quantitative and qualitative methods. This may result in important omissions, such as an adequate description of analysis [8]. However, we acknowledge that the problem of word limits may be alleviated by the increasing number of electronic journals publishing online with no word restrictions. We also recognise that the articles included in this assessment represent a limited sample of qualitative and mixed methods studies, both in number and in research fields.

### Interpretation

A possible explanation for poor reporting of research conduct and analysis procedures in mixed methods studies is that authors are not familiar with reporting criteria for qualitative research. In fact methodologists question whether one researcher can be expected to be proficient both in the qualitative and quantitative part of a mixed methods study, and suggest a division of labour to counter possible gaps in skills [9]. That mixed methods studies are judged less credible and more likely to provide thin description may relate to the reasons researchers choose a mixed methods approach – this might be to do with the value of using a combination of methods to address the research question, but equally the qualitative component might be ‘tagged on’ as a strategic decision to gain funding or credibility [20]. Another reason for the poor quality of mixed methods studies could be a lack of guidelines and training for journal reviewers and editors [21].

It is encouraging to observe a trend for better reporting of analysis procedures and more credible and rich findings in more recently published mixed methods papers. Yet we note in our small sample that reporting of research conduct (i.e. research question and rationale, sampling strategy and data collection methods) in mixed methods papers has not improved over time. This could be to do with the many uses and justifications for combining methods, and the many ways of designing and implementing mixed methods studies, which means the methods can be challenging to write up. Indeed others have observed the lack of transparency in describing the quantitative and qualitative components of mixed methods research, particularly the procedures and sophistication of analysis in the qualitative components [7]. However, the recent development of guidelines for good reporting of mixed methods studies [7] as well as criteria for appraising mixed methods studies [13] might help to improve reporting of mixed methods studies and therefore the potential for their inclusion in systematic reviews.

Given the challenges in conducting and reporting mixed methods studies, can and should they be included in systematic reviews? Our comparative analysis supports the assertion that the qualitative component of mixed methods studies is less likely to be reported well and less likely to provide the kind of thick description necessary for higher order interpretation that systematic reviews of qualitative research demand. Perhaps, then, we should be moving toward excluding papers of lower quality, focussing on research that provides credible findings with evidence of thick description [22] that illuminates the underlying conceptual linkages necessary for interpretation and generating theory. However, there are no validated methods for excluding qualitative studies from syntheses on the basis of quality. Currently review authors seem to favour an inclusive approach and systematic examination of the relative contribution of studies to the synthesis rather than exclusion of lower quality studies [23].

As well as contributing to qualitative systematic reviews, there are examples of how mixed methods studies have been included in more traditional ‘quantitative’ reviews and in mixed methods reviews that aim to answer multiple questions. An example is the ‘integrative’ review method developed by the EPPI Centre; the approach integrates data from trials (to determine effect of interventions) with data from views studies (stakeholder experiences and opinions of interventions). The views studies are usually but not always qualitative – in one example, the views studies were mainly cross-sectional surveys using questionnaires with closed and open-ended responses, or cross-sectional surveys using questionnaires combined with focus group discussions [24]. Reviews that include diverse study types help to answer questions about intervention development, appropriateness and implementation as well as questions about effect [25], and usually no one study type can answer such complex (often policy-oriented) questions. In these circumstances, where it makes sense to include mixed methods research together with other study designs in a systematic review, reviewers need quality assessment criteria that allow them to consider the extent and type of integration of methods and findings, as well as questions specific to the methods and rigor of the qualitative and quantitative components [13].

In this study we examined the types of recommendations made by studies in our sample, because we believe that qualitative health researchers should aim to make their findings relevant to policy and other decision makers. We did not examine whether recommendations originated from the study findings or from author opinion [26], but this is an area that could be incorporated into quality appraisal checklists. An important concern though, is that the studies in our sample frequently recommended ‘health education’ interventions to improve local knowledge and understanding of disease and promote behaviour change in relation to prevention and treatment of infectious disease. In these circumstances, recommendations for health education are often based on assumptions about inaccurate local knowledge and the superiority of scientific medical knowledge. Such recommendations are inappropriate when there is clear evidence that increasing knowledge, without consideration of the wider social, cultural and political context, does not lead to behaviour change [27].

### Implications for further research

We know from this comparative analysis that qualitative findings from mixed methods studies are more likely to provide thin description and less likely to be judged credible. However the findings from these studies may make important contributions in a synthesis, by confirming themes or patterns identified in studies that provide richer data or thicker description. We did not examine the conceptual contribution of studies in this analysis, but we believe this should be explored further. Another interesting question arising from this work is whether online publishing without word limits has had an effect on the quality of reporting of qualitative research. This could be done by conducting a systematic quality appraisal of a random sample of qualitative and mixed methods studies reported in the last year in electronic health and medical journals. Further work should be done to test whether the quality of reporting in mixed methods compared with qualitative only studies differs in other areas of global health, for example research on HIV/AIDS. Researchers could consider using larger sample sizes to detect any significant differences between the two study types.

## Conclusions

Mixed methods research makes an important contribution to health research in general, and could make a more substantial contribution to systematic reviews. Through our careful analysis of the quality of reporting of mixed methods and qualitative-only research, we have identified areas that deserve more attention in the conduct and reporting of mixed methods research. Our analysis has implications for authors of systematic reviews, who need to consider whether to include mixed methods research, and if so how to appraise studies that employ this design.

## Competing interests

The authors declare that they have no competing interests.

## Authors' contributions

AK and HS came up with the concept for the paper and analysed the data from the malaria systematic review. AL helped draft the initial content of the paper. SA analysed the data from the TB systematic review and drafted initial versions of the paper. All authors contributed to drafts and revisions of the paper, and read and approved the final manuscript.

## Pre-publication history

The pre-publication history for this paper can be accessed here:

http://www.biomedcentral.com/1471-2288/12/62/prepub

## Supplementary Material

Additional file 1 Characteristics of studies included in the comparative analysis (Large table; additional file). Click here for file
