# Supplementary material for: Including mixed methods research in systematic reviews: Examples from qualitative syntheses in TB and malaria control
Source: BMC Med Res Methodol. 2012 Apr 30;12:62. doi: 10.1186/1471-2288-12-62 (PMC3445834; doi:10.1186/1471-2288-12-62)
Supplement: Additional file 1 — Characteristics of studies included in the comparative analysis (Large table; additional file). [file 1471-2288-12-62-S1.doc]

## Additional file 1 - Characteristics of studies included in the comparative analysis

| **Author^1^, year** | **Country** | **Methodology category** | **Data collection methods** | **Study participants** | **Sample size** | |
| --- | --- | --- | --- | --- | --- | --- |
|  |  |  |  |  | **FGD** | **Interviews** |
| **Studies included in the systematic review on TB adherence [15]** | | | | | | |
| Mata 1985 | Honduras | Mixed methods | FGDs^2^, interviews, survey | TB patients; their relatives; health personnel; nurses & epidemiologists | FGDs (75 participants) | 90 |
| Curtis 1994 | US | Qualitative | Interviews | Drug users (women and men of different ethnic background; latino; black and white people. |  | 28 Latino  22 Black  18 White |
| Liefooghe 1995 | Pakistan | Qualitative | FGDs | Male & female hospitalized TB patients. | 6 FGDs (3 male and 3 female groups, 48 participants) |  |
| Smith 1995 | US | Mixed methods | Interviews, FGDs, record review | People who had active TB; had been exposed to TB; or who were at high risk of contracting TB in the future. | 13 FGDs | 67 |
| Johansson 1996 | Vietnam | Qualitative | Interviews, FGDs | Staff (medical doctors female nurses, nurse aide); defaulted TB patients. | FGDs (participants:  2 medical doctors, 2 male and 1 female,  3 female nurses, 2 female aide nurses) | 10 defaulted TB patients (8 male and 2 female) |
| Dick 1996 | South Africa | Qualitative | FGDs, cohort study | TB patients | 7 FGDs (8 participants each) |  |
| Menegoni 1996 | Mexico | Qualitative | Interviews | TB patients (men & women) who received treatment; most belonging to lower and middle wealth category in Mexico. |  | 33 (19 men and 14 women) |
| Nair 1997 | India | Qualitative | Interviews | TB patients (adult men & women) |  | 16 (7 men and 9 women) |
| Ellis 1997 | South Africa | Qualitative | Interviews | New TB treatment cases; retreatment cases. |  | 23 new TB patients & their households  5 re-treatment cases |
| Dick 1997 | South Africa | Mixed methods | Interviews, cohort study | patients; lay health workers; employers; health providers |  | no information |
| Ngamvithayapong 1997 | Thailand | Mixed methods | FGDs | TB patients with good adherence; patients who defaulted | 5 FGDs (2 of married men, 1 of married women, 1 of female widows – total 28 participants ) | 72 |
| Coleman 1998 | South Africa | Qualitative | Interviews | Volunteer supervisors (store keepers; other volunteers who were supervising TB patients). |  | 15 store keepers  5 lay supervisors |
| Ito 1999 | US | Qualitative | FGDs and interviews, participant observation | clinic clients (men & women; ; staff; community members; female compliant TB patients. | 1 FGD (12 compliant female participants) | 12 females,  12 males, clinic staff, passersby, |
| Johansson 1999 | Vietnam | Qualitative | FGDs | Men and women (15-60yrs) with and without previous TB experience | 16 FGDs (8-10 participants each, 4 groups per site, separate for men and women with or without TB experience) |  |
| Ngamvithayapong 2000 | Thailand | Qualitative | FGDs | Health centre staff; community members (men & women) ; people with asymptomatic HIV (men & women); male injecting drug users; HIV positive TB patients (men & women) HIV negative TB patients (men & women) | 11 FGDs (5 female groups & 6 male groups plus one group of injecting drug users without TB; 85 participants) |  |
| Khan 2000 | Pakistan | Mixed methods | Interviews, survey | New TB patients (male/female; treatment continuing, completed or defaulted; and urban and rural residence) |  | 18 men  18 women |
| Pushpananthan 2000 | Swaziland | Mixed methods | Interviews, register review, cohort study | TB patients on current treatment (men & women) |  | 48 men  30 women |
| San Sebastian 2000 | UK | Mixed methods | Interviews, survey | 24 patients undergoing preventive TB treatment (men & women) |  | 12 men  12 women |
| Edginton 2002 | South Africa | Mixed methods | FDGs, survey | Tuberculosis hospital in-patients (men & women); local traditional healers (men & women); , community members (men & women); community leaders (village indunas or male headmen); student nurses at the hospital; male manual road workers. | 14 FGDs (approx. 160 participants) |  |
| Johansson 2002 | Vietnam | Qualitative | Interviews | Patients with ongoing or a recent history of TB ( non-compliant patients, compliant patients (men & women); health care providers; medical doctors; assistant doctors (men & women) |  | Patients with ongoing or recent history of TB: 15 male 9 female  Doctors 4 male, 4 female  Assistant doctors 4 male 3 female |
| Singh 2002 | India | Mixed methods | Interviews, register review, semi-structured interviews | DOT centre patients not on the revised national TB control programme of India; TB Health Visitors. |  | 59 patients  21 Health visitors |
| Wares 2003 | Nepal | Mixed methods | Interviews, survey | completing patients & defaulting patients (men & women) from each category of supervision (Long course and short course chemotherapy that was either directly observed or unsupervised) |  | 44 men  17 women |
| Harper 2003 | Gambia | Qualitative | FGDs , interviews | Community members, including high school students, hospitalised patients and refugees, health providers and TB patients. | 40 FGDs (33 with general community) | 46 with healthcare staff  77 with successfully treated/failed/defaulted TB patients and TB patient relatives |
| Demissie 2003 | Ethiopia | Mixed methods | FGDs, interviews, cohort study | TB patients, key informants who had not had TB; cured TB patients; and patients that were TB club members | 11 FGDs with TB club members | 8 (key informants (priests) who were not TB patients, cured TB patients who were once ‘‘TB club  leaders’’ and patients who were TB club members) |
| Jaiswal 2003 | India | Mixed methods | Interviews, record and register review, community survey | TB patient defaulters |  | 40 defaulters |
| Watkins 2004 | Indonesia | Qualitative | Interviews | people with diagnosed or suspected TB |  | 5 diagnosed TB patients  6 suspected TB patients |
| Watkins 2004 | Indonesia | Qualitative | Interviews, FGDs | Clinic staff involved in delivery of TB treatment; 19 doctors, 13 nurses, 3 healthcare workers, 2 laboratory workers – 16 were female | 20 in 9 FGDs | 17 |
| Sanou 2004 | Burkina Faso | Qualitative | FGDs, interviews | Male and female TB patients, community representatives, members of the health centre management committee, traditional healers and health professionals. health centre management committee members, female community members and male community members. | 28 FGDs | 68 |
| Marra 2004 | Canada | Qualitative | FGDs, interviews | English, Cantonese, and Punjabi-speaking subjects with active TB receiving treatment - 39 in total | people with active TB - no information | People attending TB clinic |
| Joseph 2004 | US | Qualitative | FGDs | Clinical and nonclinical staff at hospitals and health departments. | 106 in 16 FGDs |  |
| Greene 2004 | Bolivia | Qualitative | Interviews | Non-adherent TB patients |  | 5 non-adherent TB patients |
| Coreil 2004 | US | Qualitative | FGDs | Men and women of Haitian origin | 5 FGDS (stratified by age and sex) |  |
| Khan 2005 | Pakistan | Mixed methods | FGDs, interviews, survey | Male and female TB patients part of a larger trial; family members, health facility staff and community health workers. | 8 FGDs (4 male, 4 female – observed by health facility staff, community health care worker, family member or self administered) | 32 successfully treated  7 defaulted  (of which 18 female, 21 male) |
| Estcott 2005 | Swaziland | Qualitative | Interviews | TB patients, RHMs and family treatment supporters |  | 10 patients  3 family treatment supporters  10 community health workers  7 clinic nurses  2 community health worker coordinators  6 TB team members (in a group)  4 hospital doctors |
| Rowe 2005 | South Africa | Qualitative | Interviews | Male and female TB patients and health care workers who routinely  administered TB treatment at the hospital | 18 patients in three groups – eligible for treatment, completed treatment and interrupted treatment | 2 healthcare workers |
| **Studies included in the systematic review on malaria in pregnancy [16]** | | | | | |  |
| Helitzer-Allen 1992 | Malawi | Mixed method | Interviewer-administered questionnaire; in depth interviews; FGDs; | Pregnant women; women who had recently given birth; chief & village headmen; husbands of pregnant women; TBAs; health workers; traditional advisors; traditional healers | 24 FGDs | 160 |
| Okonofua 1992 | Nigeria | Mixed method | Survey; FGDs | Fathers and mothers of adolescents; pregnant & non pregnant adolescents; TBAs | 3 FGDs (8-16 participants each) |  |
| Kengeya-Kayondo 1994 | Uganda | Qualitative | FGDs; semi-structured interviews; key informant interviews | ANC clinic attendees; women bringing children for vaccinations; members of women’s clubs; female outpatients; females escorting children with malaria; key informants (mothers) | 5 FGDs (42 participants) | 395 semi-structured interviews  64 mothers |
| Ghebreyesus 1996 | Ethiopia | Mixed method | FGDs | Community members; CHWs | No information |  |
| Ndyomugyenyi 1998 | Uganda | Mixed method | FDGs; in depth interviews; structured interviewer-administered questionnaire | Pregnant women of different parity; health personnel from formal health system; TBAs | 10 FGDs | 4 TBAs  4 health personnel |
| Rashed 1999 | Benin | Mixed method | Household questionnaire; FGDs; semi structured interviews | Men & women of reproductive age who are parents ; informants (community elders; healers; health care providers; | 23 FGDs | 16 |
| Dulhunty 2000 | Solomon Islands | Mixed method | Structured interview questionnaire; FGDs; structured observations | Women and men | No information |  |
| Baume 2001 | Senegal | Qualitative | Consumer interview; FGDs | Parents of children under 5 (some net owners, some not); | 8 FGDs | 40 consumers |
| Baume 2001 | Uganda | Qualitative | Consumer interview; FGDs; product demonstration; key informant interview | Parents of children under 5 (some net owners, some not); key informants (traders of insect control products from a range of outlets) | 10 FGDs | 50 consumers  32 traders |
| Baume 2001 | Zambia | Qualitative | Consumer interview; FGDs; product demonstration; key informant interview | Parents of children under 5 (some net owners, some not); key informants (traders of insect control products from a range of outlets) | 10 FGDs | 50 consumers  25 traders |
| Baume 2001 | Nigeria | Qualitative | Consumer interview; FGDs; product demonstrations; key informant interviews |  | 10 FGDs | 51 consumers  26 traders |
| Ashwood-Smith 2002 | Malawi | Mixed method | Researcher administered survey (with open ended questions) | Health personnel; antenatal mothers |  | 41 health personnel  287 antenatal mothers |
| Miaffo 2004 | Burkina Faso | Mixed method | FGDs; key informant interviews; structured questionnaire | Pregnant women ANC users; husbands of ANC users; pregnant women ANC non-users; key informants (health workers; TBAs; women group leaders) | 6 FGDs (6-12 participants each) | 40 key informants |
| Muula 2004 | Malawi | Qualitative | In depth interviews | Health workers in public health facilities |  | 21 |
| Kikumbih 2005 | Tanzania | Mixed method | Household survey; costing study; FGDs | Adult men; adult women; women with children under 5 | No information (8-10 participants each) |  |
| Mubyazi 2005 | Tanzania | Qualitative | FGDs; in depth interviews | Pregnant women; key informants; dispensary staff | 6 FGDs (8-10 participants each) | 11 key informants |
| Launiala 2006 | Malawi | Mixed method | In depth interviews; key informant interviews; KAP survey | Women of reproductive age; key informants (TBAs, traditional advisors, traditional healer, men) | 8 FGDs | 34 women  8 key informants |
| Mbonye 2006a | Uganda | Qualitative | FGDs; key informant interviews; | pregnant women; non-pregnant women; adolescent girls; men; opinion leaders (local council officials; elderly midwives; retired women teachers; drug shop owners; TBAs) | 10 FGDs (90 participants) | 40 key informants |
| Mbonye 2006b | Uganda | Qualitative | FGDs; key informant interviews | pregnant women; non-pregnant women; adolescent girls; men; opinion leaders (local council officials; elderly midwives; retired women teachers; drug shop owners; TBAs) | 10 FGDs (90 participants) | 40 key informants |
| Mbonye 2006c | Uganda | Qualitative | FGDs; Key informant interviews | pregnant women; non-pregnant women; adolescent girls; men; opinion leaders (local council officials; elderly midwives; retired women teachers; drug shop owners; TBAs) | 10 FGDs (90 participants) | 40 key informants |
| Ahorlu 2007 | Ghana | Qualitative | Free listing, participatory mapping, FGDs, interviews | Men & women of different ages; key informants (chiefs & elders; women's leaders; pregnant women; caretakers of children under 5) | 4 FGDs | 14 |
| Kweku 2007 | Ghana | Mixed method | Tool to monitor voucher distribution; semi-structured questionnaires with open questions | Staff involved in voucher distribution; midwives in ANC services; retailers within ANC catchment; pregnant women |  | Round 1: 346  Round 2: 323  Round 3: 288 |
| Launiala 2007 | Malawi | Mixed method | FGDs; in depth interviews; participant observation; KAP survey | Women of reproductive age | 8 FGDs | 34 |
| Mbonye 2007a | Uganda | Mixed method | Survey with open ended questions; key informant interviews | Women who participated in community based IPT delivery intervention study; women who did not participate; key informants (resource persons, health workers, opinion leaders) |  | 108 women  60 key informants |
| Mbonye 2007b | Uganda | Mixed method | Survey with open ended questions; key informant interviews | Health workers; women who participated in community based IPT delivery intervention study; opinion leaders |  | 60 key informants |
| Appleyard 2008 | Solomon Islands | Mixed method | FGDs; semi structured interviews; observation; structured questionnaire | ANC staff; malaria officers; pregnant women | 2 FGDs | 5 |
| Belay 2008 | Ethiopia | Mixed method | FGDs ; structured interview questionnaire | Pregnant women; CHW's^3^; male heads of households; community leaders | 4 FGDs (6-12 participants each) |  |
| Haileselassie 2008 | Ethiopia | Mixed method | Structured interviewer-administered questionnaire; FGDs | Women and men | 4 FGDs |  |
| Idowu 2008 | Nigeria | Mixed method | Researcher-administered questionnaire; FGDs | Primigravids; multigravids; teenagers and non-teenagers; women of different educational status | No information |  |
| Mubyazi 2008 | Tanzania | Qualitative | In depth interviews; telephone interviews; personal communications | NMCP^4^ officers; MoH officers; private health care providers; |  | 5 NMCP officers  2 MoH officers  No information on private providers |
| Mushi 2008 | Tanzania | Mixed method | FGDs; in depth interviews | Community resource persons; mothers of babies and pregnant women at a) village centres; b) RCH clinics; c) outlying hamlets | 52 FGDs | 8 |
| Stokes 2008 | Gambia | Qualitative | FGDs; in depth interviews | Nuliparious & parous women; men regarded as senior; TBAs | 16 FGDs (83 participants) | 41 |
| Tolhurst 2008 | Malawi | Qualitative | FGDs; critical incidence interviews; key informant interviews | Mothers; fathers; grandmothers; women who have experienced pre-term birth; key informants (TBAs; clinic health workers; traditional healers) | 17 FGDs (177 participants) | 13 key informants |
| Baume 2009 | Ethiopia | Mixed method | Household survey with integrated open ended questions; observation | Households (husband or wife); most often was female head of household |  | 857 household interviews |
| Brabin 2009 | Gambia | Qualitative | In depth interviews; FGDs | Nuliparious & parous women; men regarded as senior; TBAs | 16 FGDs | 41 |
| Beiersmann 2010 | Burkina Faso | Qualitative | FGDs; individual interviews | Head nurses; ANC providers; key informants (pharmacists; director social marketing; social marketing promoters; wholesaler; rural shopkeepers; director National Malaria Programme) | 4 FGDs | 11 key informants |
| Chuma 2010 | Kenya | Mixed method | Household survey; FGDs; semi-structured interviews | ITN suppliers (retailers; public health facilities; NGO’s); men & women of reproductive age; older men & women; key informants | 24 FGDs | 27 retail outlets  32 public health facilities  11 NGOs |
| Howard 2010 | Afghanistan | Mixed method | FGDs; in depth interviews; key informant interviews; household survey | Men & women from ITN owning and non-owning households; key informants (clinic staff; informal providers; ITN implementers) | 15 FGDs (82 men; 40 women) | 30  7 key informants |
| Kolazinski 2010 | Uganda | Mixed method | Household survey; FGDs; interviews | Men & women who a) neither received a net or information about use; b) received a net & information; c) received a net but did not use it or did not retain it | 20 FGDs | 17 with men & women who received a net but did not use it or did not retain it |
| Mbonye 2010 | Uganda | Mixed method | Household survey; survey of midwives; key informant interviews | Key informants (civic leaders; HIV positive women; local council officials; private midwives; midwives in public facilities; teachers; pregnant and non pregnant women) |  | 66 |
| Mubyazi 2010 | Tanzania | Qualitative | FGDs; semi-structured exit interviews; observation | Village level: pregnant women; mothers with infants; facility level exit interviews: pregnant women following ANC visit | 24 FGDs (6-12 participants each) | 823 exit interviews |
| Sabin 2010 | India | Qualitative | In depth interviews; FGDs | Pregnant women older than 15yrs; | 6 FGDs (73 participants) | 32 |
| Smith 2010 | Ghana | Qualitative | FGDs; | Participants in an RCT( pregnant women from each study arm of the RCT) | 12 FGDs |  |

^1^ Last name of first author; ^2^ Focus Group Discussions; ^3^ Community Health Workers; ^4^ National Malaria Control Programme
